# An efficient protocol for studying human pluripotent stem cell-derived myotube senescence

**DOI:** 10.52601/bpr.2023.230013

**Published:** 2023-10-31

**Authors:** Qian Zhao, Ying Jing, Shuai Ma, Weiqi Zhang, Jing Qu, Si Wang, Guang-Hui Liu

**Affiliations:** 1 Advanced Innovation Center for Human Brain Protection, National Clinical Research Center for Geriatric Disorders, Xuanwu Hospital Capital Medical University, Beijing 100053, China; 2 Aging Translational Medicine Center, International Center for Aging and Cancer, Beijing Municipal Geriatric Medical Research Center, Xuanwu Hospital, Capital Medical University, Beijing 100053, China; 3 State Key Laboratory of Stem Cell and Reproductive Biology, Institute of Zoology, Chinese Academy of Sciences, Beijing 100101, China; 4 State Key Laboratory of Membrane Biology, Institute of Zoology, Chinese Academy of Sciences, Beijing 100101, China; 5 University of Chinese Academy of Sciences, Beijing 100049, China; 6 Beijing Institute for Stem Cell and Regenerative Medicine, Beijing, 100101, China; 7 CAS Key Laboratory of Genomic and Precision Medicine, Beijing Institute of Genomics and China National Center for Bioinformation, Chinese Academy of Sciences, Beijing 100101, China; 8 Institute for Stem Cell and Regeneration, CAS, Beijing 100101, China; 9 Sino-Danish College, University of Chinese Academy of Sciences, Beijing 101408, China; 10 Sino-Danish Center for Education and Research, Beijing 101408, China

**Keywords:** Human pluripotent stem cell, Myotube, Senescence, Differentiation, *In vitro*

## Abstract

Sarcopenia, an age-related skeletal muscle condition characterized by a progressive decline in muscle mass and function, is linked to increased vulnerability, a higher likelihood of falls, and higher mortality rates in older individuals. A comprehensive understanding of the intricate mechanisms driving skeletal muscle aging is of great significance in both scientific and clinical fields. Consequently, myotube models that facilitate studying regulatory mechanisms underlying skeletal muscle aging are important tools required to advance intervention strategies against skeletal muscle aging and associated disorders. Here, we provide a detailed protocol to generate human pluripotent stem cells-derived myotubes and describe their applications in aging studies, as well as a troubleshooting for potential problems. Overall, this protocol serves as a valuable methodological reference for exploring the role and mechanism of genes involved in skeletal muscle aging.

## INTRODUCTION

Skeletal muscles, constituting approximately 40% of the total body mass, play a crucial role in supporting body posture, integrating movement across various body regions, safeguarding joints, and so on (Frontera and Ochala 2015). In skeletal muscle, muscle cells fuse to create long or connected multinucleated myofibers enclosed by the sarcolemma, the excitable cell membrane which connects muscle cells with the basement membrane to form strong contractile muscle fibers. Although myofibers are responsible for the major function of skeletal muscle, multiple interstitial cells including fibro/adipogenic precursors (FAPs), endothelial cells and immune cells make important contributions to the muscle tissue microenvironment (De Micheli *et al.* 2020; Dell'Orso *et al.* 2019; Jing *et al.* 2023).

Aging is characterized by a progressive deterioration in the preservation of tissue homeostasis, organ functionality, and stress response (López-Otín *et al.* 2023). In skeletal muscle, this deterioration or sarcopenia, which refers to the loss of skeletal muscle mass and strength associated with aging, notably decreases mobility and balance in the elderly (Larsson *et al.* 2019), as well as increases overall frailty and risk of falls, disability, and potential mortality. Recent single cell/nucleus RNA sequencing of primate aging found that skeletal muscle fibers have a higher susceptibility to aging, which is mainly manifested by the higher transcriptional noise in muscle fibers and increased numbers of differentially expressed genes in aging (Jing *et al.* 2023). To investigate the underlying mechanisms of skeletal muscle aging, a range of cellular and animal models are required.

To date, a variety of cell models have been utilized to investigate skeletal muscle aging. For instance, a pioneering study reported that primary muscle cells could be isolated and cultured from skeletal muscle tissues of experimental animal models or humans (Musarò and Carosio 2017; Spinazzola and Gussoni 2017; Yin *et al.* 2018), and that such cultures mimic *in vivo* skeletal muscle biology and enable the study of mechanisms underlying skeletal muscle aging. However, the workflow to obtain primary cells is complex, and their poor expansion potential greatly limits experimental feasibility. More importantly, ethical limitations make it very challenging to obtain enough disease-free and age-matched skeletal muscle from biopsies. Therefore, skeletal muscle cell lines or myoblast cell lines, such as C2C12 in mice and Hu5/KD3 in humans, that are derived from immortalized primary cells capable of indefinite expansion, are widely used (Nagashima *et al.* 2020; Reynolds *et al.* 2021). Skeletal muscle cell lines offer convenience for experimental manipulation, but could not faithfully mimic the functional aspects of skeletal muscle aging *in vivo*. Therefore, human pluripotent stem cells-derived skeletal muscle cells have been proposed as valuable models for generating a large amount of cell materials to study human skeletal muscle aging (Shahriyari *et al.* 2022; Steele-Stallard *et al.* 2018). In addition, embryonic stem cells can easily be gene-edited to introduce disease drivers capable of mimicking human diseases, thereby enabling mechanistic and quantitative profiling studies that may offer a more precise understanding of the mechanisms underlying skeletal muscle-related disorders.

Here, we present a workflow for generation and usage of human myotube model to study skeletal muscle aging, which includes the differentiation of human pluripotent stem cell-derived myogenic progenitor cells into myotubes, as well as the generation and characterization of senescent myotubes. Additionally, we offer guidelines and suggestions to enhance the differentiation efficiency and facilitate the interpretation of the obtained results. Overall, this protocol provides a detailed procedure for investigating molecular mechanisms underlying human myofiber aging *in vitro*.

## SUMMARIZED PROCEDURE

**Figure 1:**
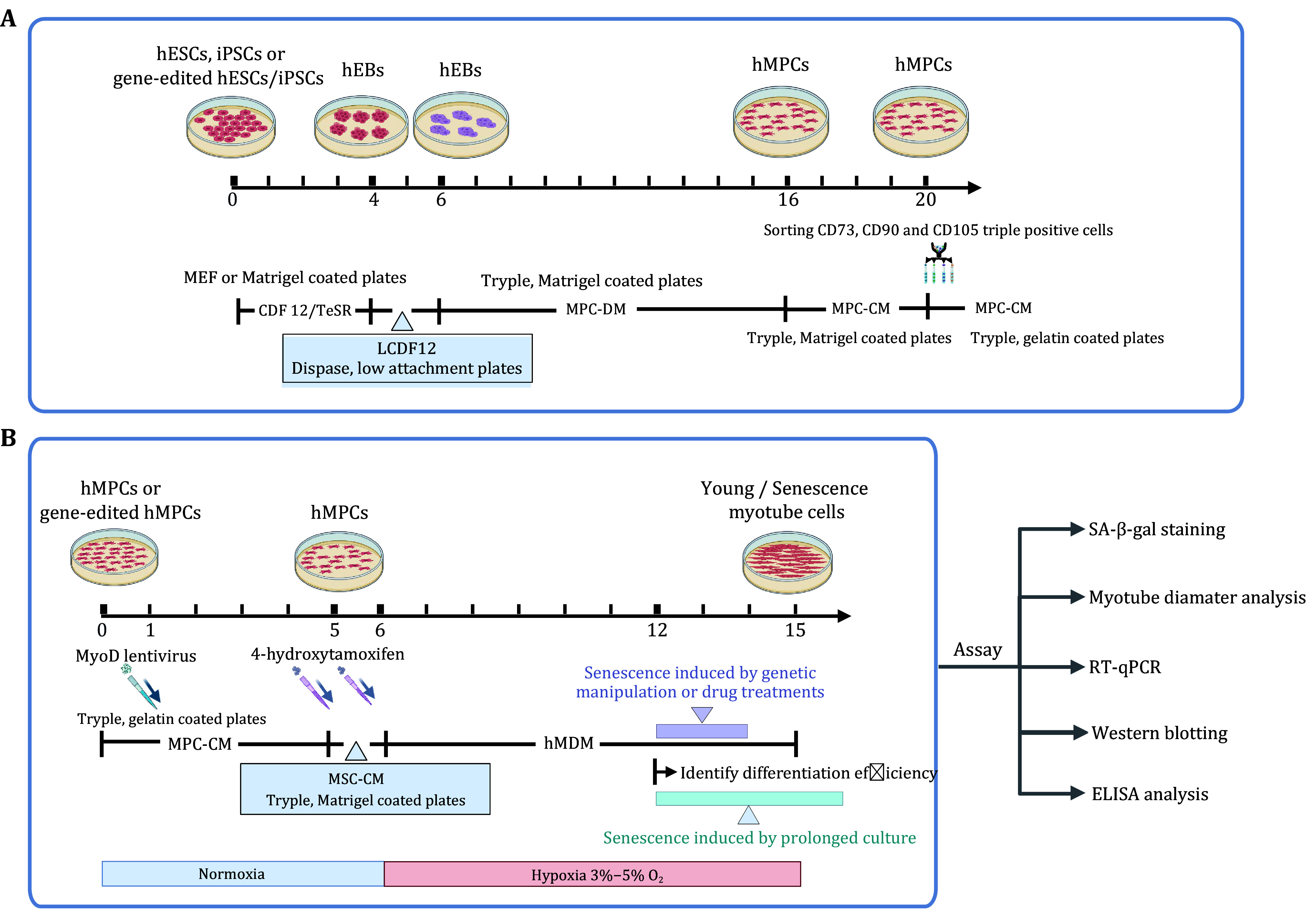
Workflow for human myotube differentiation and its application in aging study. **A** Schematic diagram showing the first differentiation step, *i*.*e*., differentiation of pluripotent stem cells into hMPCs. **B** Schematic diagram showing the second differentiation step, *i*.e., differentiation of hMPCs into myotubes, and analysis of senescence-associated phenotypes

The protocol consists of three sections. The first section provides the detailed procedure for differentiating human pluripotent stem cells into human myogenic progenitor cells (hMPCs). The pluripotent stem cells including human embryonic stem cells (hESCs), pluripotent stem cells (iPSCs) and hESCs/iPSCs generated by gene editing technology. Importantly, before starting differentiation, assessing pluripotency is a prerequisite for an effective outcome. Pluripotency is assessed through expression analysis of pluripotency genes (*OCT4*, *SOX2*, *NANOG*) and examination of teratoma formation *in vivo*, the gold standard assay for testing the differentiation potential of human pluripotent stem (Pan and Thomson 2007). In the second section, we provide a detailed guideline for differentiating hMPCs into human myotubes by introducing the core transcription factor *MYOD1*, which both induces cell cycle arrest and activates the myogenic program (Maffioletti *et al.* 2015). In this part, we describe how we transduce hMPCs with the lentiviral vector carrying *Myod1* cDNA fused with the tamoxifen-regulated estrogen receptor (MyoD-ER(T)) cassette, thus allowing inducible MYOD protein expression in the presence of 4-hydroxytamoxifen. In the third section, we describe in detail different treatments and assays for induction and characterization of myotube senescence *in vitro*. To induce myotube cell senescence, we perform gene editing-mediated gene manipulation, prolonged culture, siRNA-mediated gene silencing or drug treatments. Senescence-associated phenotypes can be assessed by analysis of the myotube diameter, the efficiency of nuclear fusion, SA-β-gal positivity, and the expression levels of aging hallmarks, such as *CDKN1A, CDKN2A, IL6, CXCL8*. A schematic for the workflow is shown in Fig. 1.

## MATERIALS AND EQUIPMENTMATERIALS

### Reagents

• DMEM/F12 (Gibco, Cat. #11330-032)

• GlutaMAX™-I (Gibco, Cat. #35050-061)

• MEM NEAA (Gibco, Cat. #11140-050)

• KnockOut^TM^ SR-Muti-Species (Gibco, Cat. #A31815-02)

• Penicillin Streptomycin (Gibco, Cat. #15140-122)

• 2-Mercaptoethanol (Gibco, Cat. #21985-023)

• bFGF (Joint Protein Central, Cat. #BBI-EXP-002)

• Fetal Bovine Serum (Gibco, Cat. #26170043)

• Dispase (Gibco, Cat. #17105041)

• TrypLE™ Express Enzyme (Gibco, Cat. #12604-021)

• MEMα + GlutaMAX™-I (Gibco, Cat. #132571-036)

• DMEM/High glucose (HyClone, Cat. #SH30243.01)

• Plasmocin™ Treatment (InvivoGen, Cat. #ant-mpt)

• Phosphate buffered saline (Sigma, Cat. #P4417)

• Horse serum (Gibco, Cat. #16050122)

• Collagenase-type Ⅳ (Gibco, Cat. #17105041)

• Polybrene (Sigma, Cat. #107689)

• Gelatin from cold water fish skin (Sigma, Cat. #G7041)

• 4-hydroxytamoxifen (Sigma, Cat. #H7904)

• TGF-β (Humanzyme, Cat. #HST-TB1-1000)

• 0.5% growth factor reduced Matrigel (BD Bioscience, Cat. #354230)

• Ultra-low attachment 6-well plate (Corning, Cat. #07-200-601)

• pLv-CMV-MyoD-ER(T) vector (Addgene, Cat. #26809)

• MyoD-ER(T) integrating lentiviral vector carrying the pLv-CMV-MyoD-ER(T) plasmid

• Anti CD73-PE antibody (BD Bioscience, Cat. #550257)

• Anti CD90-FITC antibody (BD Bioscience, Cat. #555595)

• Anti CD105-APC antibody (BD Bioscience, Cat. #17105742)

• Hoechst 33342 (Invitrogen, Cat. #H3570)

• Anti MyHC antibody (DSHB, Cat. #MF20)

### Equipment

• Centrifuge (Thermo Fisher Scientific, Cat. #MicroCL 21R)

• Flow cytometer (BD, FACSAria III)

• Cell incubator, 5% CO_2_, 3%–5% O_2_ (Thermofosher, 150i)

• Cell incubator, 5% CO_2_, 21% O_2_ (Thermofosher, 150i)

### Reagent Setup

The compositions needed for the reagent setup, as well as their volume and final concentration, are listed in Tables 1–6.

**Table 1:** hESC culture medium (CDF12)

Composition	Volume	Final concentration
DMEM/F12	385 mL	
KnockOut^TM^ SR-Multi-Species	100 mL	20% (*v*/*v*)
GlutaMAX^TM^-1	5 mL	2 mmol/L
MEM NEAA	5 mL	0.1 mmol/L
Penicillin Streptomycin	5 mL	1% (*v*/*v*)
2-Mercaptoethanol	0.5 mL	0.1% (*v*/*v*)
bFGF (50 μg/mL)	100 μL	10 ng/mL
Plasmocin^TM^ Treatment	50 μL	0.01% (*v*/*v*)

**Table 2:** Low bFGF ESC culture medium (LCDF12)

Composition	Volume	Final concentration
DMEM/F12	385 mL	
KnockOut^TM^ SR-Multi-Species	100 mL	20% (*v*/*v*)
GlutaMAX^TM^-1	5 mL	2 mmol/L
MEM NEAA	5 mL	0.1 mmol/L
Penicillin Streptomycin	5 mL	1% (*v*/*v*)
2-Mercaptoethanol	0.5 mL	0.1% (*v*/*v*)
bFGF (50 μg/mL)	50 μL	5 ng/mL
Plasmocin^TM^ Treatment	50 μL	0.01% (*v*/*v*)

**Table 3:** hMPC differentiation medium (MPC-DM)

Composition	Volume	Final concentration
MEMα + GlutaMAX^TM^-1	44 mL	
Fetal bovine serum	5 mL	10% (v/v)
TGF-β (50 μg/mL)	5 μL	5 μg/mL
bFGF (50 μg/mL)	10 μL	10 ng/mL
MEM NEAA	0.5 mL	1% (*v*/*v*)
Penicillin Streptomycin	0.5 mL	1% (*v*/*v*)
Plasmocin^TM^ Treatment	5 μL	0.01% (*v*/*v*)

**Table 4:** hMPC culture medium (MPC-CM)

Composition	Volume	Final concentration
MEMα + GlutaMAX^TM^-1	440 mL	
Fetal bovine serum	50 mL	10% (*v*/*v*)
bFGF (50 μg/mL)	100 μL	10 ng/mL
MEM NEAA	5 mL	1% (*v*/*v*)
Penicillin Streptomycin	5 mL	1% (*v*/*v*)
Plasmocin^TM^ Treatment	50 μL	0.01% (*v*/*v*)

**Table 5:** FACS buffer

Composition	Volume	Final concentration
PBS	44 mL	
Fetal bovine serum	5 mL	10% (*v*/*v*)
Plasmocin ^TM^ Treatment	0.05 μL	0.1% (*v*/*v*)
Penicillin Streptomycin	1 mL	2% (*v*/*v*)

**Table 6:** hMyoblast cell differentiation medium (MDM)

Composition	Volume	Final concentration
High glucose DMEM	475 mL	
Horse serum	10 mL	2% (*v*/*v*)
GlutaMAX^TM^-1	5 mL	2 mmol/L
MEM NEAA	5 mL	0.1 mmol/L
Penicillin Streptomycin	5 mL	1% (*v*/*v*)
Plasmocin^TM^ Treatment	50 μL	0.01% (*v*/*v*)

## PROCEDURE

### Step 1: From pluripotent stem cells to hMPCs [TIMING ~20 days]

**Figure 2:**
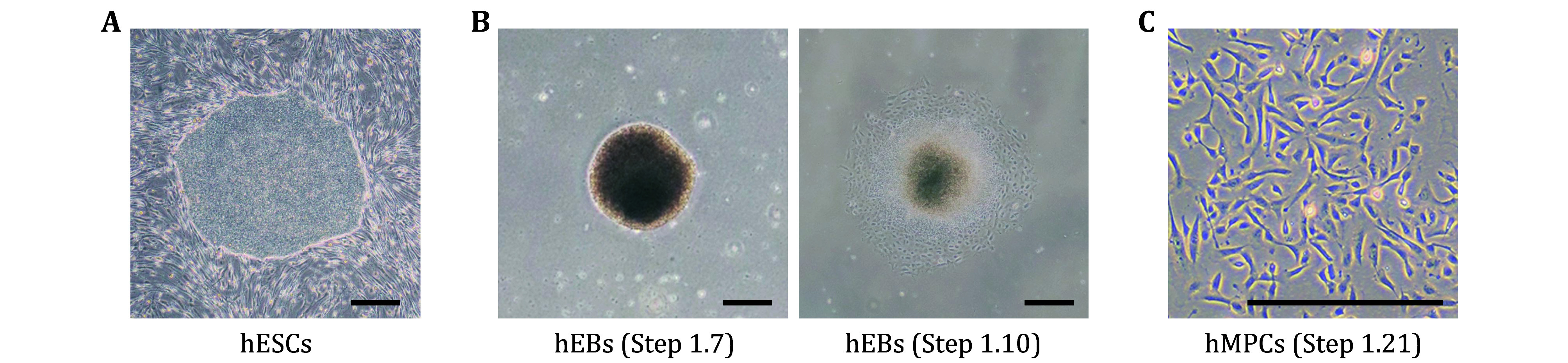
Differentiation of pluripotent stem cells into hMPCs. **A** The representative image of hESCs with a good growth status. **B** Representative images of morphological characteristics of 2-day hEB morphology in Step 1.7 (left) and 3-day hEB in Step 1.10. **C** The representative image of P2 hMPCs sorted by CD73, CD90, and CD105-triple positivity. Scale bars, 400 μm

Step 1.1: Maintain hESCs on Mitomycin C-treated mouse embryonic fibroblasts (MEFs) in CDF12 medium (Fig. 2A) or on Matrigel (BD Biosciences)-coated plates in mTeSR medium, up to a maximum of 70 passages.

Step 1.2: Three to four days after hESCs seeding, discard the supernatant and rinse twice with DMEM/F12 medium.

Step 1.3: Add 1 mL dispase (0.5 mg/mL) for one well of the 6-well plate and incubate at 37 °C for about 20 min until 60%–80% of the clones have been detached.

**[CRITICAL STEP]** Don’t keep the clones in the dissociation medium for more than 30 min as doing so greatly decreases cell survival and compromises the following steps.

Step 1.4: Collect the dissociation medium with the clones, and add 2 mL CDF12 medium.

Step 1.5: Let stand for 4 min until the clones sink to the bottom, and discard the supernatant.

Step 1.6: Add 6 mL CDF12 medium, and then repeat Step 1.5 twice to dilute the residual dispase, and then resuspend in 2 mL LCDF12 medium.

Step 1.7: Plate the clones on ultra-low attachment 6-well plates to form human embryoid bodies (hEBs), as shown in Fig. 2B.

**[CRITICAL STEP]** Clones should be intact and of similar size.

Step 1.8: On the next day, change half of the culture medium with LCDF12 medium and prepare a 6-well dish coated with 0.5% growth factor reduced Matrigel.

Step 1.9: On the next day, transfer 10–14 hEBs to 6-well plates pre-coated with Matrigel. The next day, change the medium to hMPC-DM.

**[CRITICAL STEP]** hEBs should be of similar size and intact under the stereoscope.

Step 1.10: Change the MPC-DM medium every other day for about ten days until fibroblast-like populations emerge (Fig. 2B).

Step 1.11: Wash the cells twice with PBS. Add 1 mL TrypLE™ Express Enzyme to each well and incubate at 37 °C for about 10 min until 80% of the cells are digested.

Step 1.12: Add 2 mL MPC-DM and collect the cell suspension. Centrifuge the cell suspension at 300 rcf at room temperature for 5 min.

Step 1.13: Discard the supernatant and resuspend the pellet in MPC-CM. Plate 2 × 10^5^ cells per 10-cm dish pre-coated with 0.5% growth factor-reduced Matrigel, and then replace the medium each other day with MPC-CM medium for about 5 d or until cells are reaching 90% confluence.

Step 1.14: Wash once with 1 mL PBS, and digest the cells with 3 mL TrypLE™ Express Enzyme at 37 °C for 2–3 min, neutralize the digestion with 6 mL MPC-CM, collect and centrifuge the cell suspension at 300 rcf at room temperature for 5 min.

Step 1.15: Wash the pellet with 8 mL PBS, resuspend in medium and count cell numbers, and then resuspend the pellet in 100 μL FACS buffer per 10^6^ cells.

Step 1.16: Take out 3 × 10^5^ cells as the negative control for FACS, and add 300 μL FACS buffer in the flow tube. Then add antibody mixture including 1 μL CD73-PE, 0.5 μL CD90-FITC (488), 0.5 μL CD105-APC per 100 μL, and incubate at 4 °C for 30 min in the dark. Gently pipette the cell suspension every 5 min.

Step 1.17: Add 10 mL FACS buffer to wash, and centrifuge the cells and then discard the supernatant.

Step 1.18: Add another 10 mL FACS buffer to washand centrifuge the cells and then discard the supernatant, add 400–500 μL FACS buffer to resuspend and filter the cells through a 40-μm cell strainer, and then transfer them into the flow tube.

Step 1.19: Sort CD73, CD90, and CD105-triple positive cells into the collection tube by Flow cytometer.

**[CRITICAL STEP]** Select triple positive cells, and maintain cell viability in a sterile environment during flow sorting.

Step 1.20: Centrifuge the sorted cell suspension at 300 rcf at 4 °C for 3 min, and plate 1–2 × 10^5^ cells in hMPC-CM per well of 6-well plates, recording passage 0 (P0). Replace the medium every other day.

Step 1.21: When reaching 95% confluence, hMPCs can be passaged, expanded, and cryopreserved for further use (Fig. 2C).

### Step 2: Induction of myogenic differentiation [TIMING ~15 days]

Step 2.1: Expand or recover P1–P3 hMPCs on 0.1% gelatin pre-coated plates, and culture with MPC-CM until the density is reaching 80% confluence.

**[CRITICAL STEP]** P1–P3 hMPCs are recommended to use given their superior differentiation potential.

Step 2.2: Infect cells with the lentiviral vector carrying MyoD1 fused with the estrogen receptor (MyoD-ER(T)) with a multiplicity of infection (MOI) < 5.

**[CRITICAL STEP]** Package the lentiviral vector and titrate the MOI to determine the highest differentiation efficiency-to-MOI rate. The differentiation efficiency is assessed by MyHC-positive myotubes. In general, the efficiency is over 90% after lentivirus transduction.

**Figure 3:**
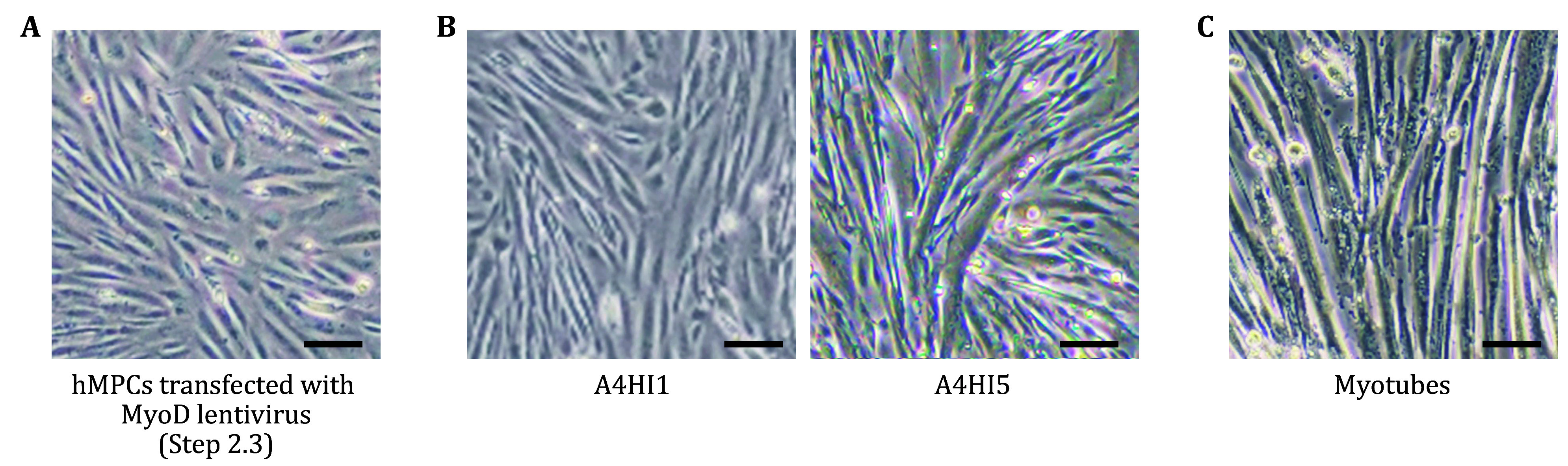
Differentiation of hMPCs into myotubes. **A** hMPCs after transfected with MyoD lentivirus for 72 h in Step 2.3. **B** Representative images of MyoD-overexpressing hMPCs after 4-hydroxytamoxifen induction (A4HI) for 1 and 5 d. **C** Myotubes induced from hMPCs after 4-hydroxytamoxifen induction for 9 d. Scale bars, 50 μm

Step 2.3: Replace the medium with MPC-CM 24 h after lentivirus transduction, and continue culture for another 2 d (Fig. 3A).

Step 2.4: Prepare a 6-well dish coated with 0.5% growth factor reduced Matrigel one day before digestion.

Step 2.5: Wash the dish twice with PBS. Add 1 mL TrypLE™ Express Enzyme for one well of the 6-well plate, and incubate at 37 °C for about 3 min until 80% of the cells are digested.

Step 2.6: Add 2 mL MPC-CM, and collect the cell suspension in a 15 mL tube. Centrifuge the cell suspension at 300 rcf at room temperature for 5 min.

Step 2.7: Discard the supernatant and resuspend the pellet in MPC-CM. Plate 2 × 10^5^ cells per well of a 6-well dish pre-coated with 0.5% growth factor reduced Matrigel.

Step 2.8: Replace the medium with MPC-CM containing 1 μmol/L 4-hydroxytamoxifen (which drives activation of the myogenesis regulator *Myod1*) at 37 °C with 5% CO_2_ and 3%–5% O_2_ after 24 h. From now on, cells are cultured in hypoxic conditions (5% CO_2_ and 3%–5% O_2_) (Fig. 3B).

Step 2.9: Change the culture medium the next day with MDM containing 1 μmol/L 4-hydroxytamoxifen (Fig. 3B).

Step 2.10: Replace the MDM medium 2 d later and change the MDM medium each other day for about 6 d (Fig. 3C).

Step 2.11: Evaluate the differential efficiency by immunofluorescence staining of MyHC (a standard marker for myotube formation) (Maffioletti *et al.* 2015). If the efficiency reaches more than 90%, further analyses can be carried out.

### Step 3: Treatments and assays for myotube senescence

*Step 3.1*:* Myotube senescence induced by genetic manipulation*

(A) Gene editing can be performed in hESCs or iPSCs. Alternatively, gene editing can be conducted at the hMPCs stage.

(B) Differentiate gene-edited hESCs or iPSCs into hMPCs according to the above protocol. Then, induce myogenic differentiation from gene-edited hESCs/iPSCs derived hMPC or directly from gene-edited hMPCs by transfecting with MyoD lentivirus according to the above protocol.

(C) Induce hMPC into myotubes following Step 2.

**[CRITICAL STEP]** The differentiation efficiency of myotube cells derived from gene-edited hESCs/iPSCs or directly gene-edited hMPCs needs to be evaluated before myotube senescence analyses.

(D) On days 5–6 after myotube differentiation initiating, myotube senescence phenotypes would be assessed.

*Step 3.2*:* Myotube senescence induced by prolonged culture*

Prolonged culture of human myotubes serves as a convenient model for studying skeletal muscle aging *in vitro*.

(A) Induce hESCs or iPSCs differentiation into maturated myotube following the above-mentioned Steps 1 and 2.

(B) Collect induced differentiated cell samples on days 6, 10 and 14 after myotube differentiation to examine aging-related phenotypes.

*Step 3.3*:* Myotube senescence induced by siRNA-mediated gene silencing*

(A) Purchase siRNA molecules. For one well of the 6-well plate, add 6 μL Lipofectamine RNAiMAX reagent fully mixed with 125 μL Opti-MEM medium.

(B) Dilute siRNA in 125 μL Opti-MEM medium and then mix fully.

(C) Add diluted siRNA duplexes to the diluted Lipofectamine RNAiMAX Reagent (1:1 ratio) and then incubate for 10–15 min at room temperature.

(D) Add siRNA-Lipofectamine RNAiMAX complex solution to matured myotubes and replace with fresh culture medium after 6–8 h.

(E) 48 h after transfection, collect cells and measure the mRNA levels of target genes by RT-qPCR.

(G) 4 d after transfection, collect myotubes for immunofluorescence staining to detect the knockdown efficiency of target genes and to conduct phenotype characterization.

*Step 3.4*:* Reference dosing time points in long-term cultured myotubes*

(A) On days 5–6 after initiation of myotube differentiation, treat matured myotubes with the small molecule drug.

(B) For about 4 d, add the small molecule drug freshly to the medium each time when the medium is changed.

*Step 3.5*:* Assays for characterization of myotube senescence*

Step 3.5.1: SA-β-gal staining

(A) Wash the cultured myotubes with PBS for two times, and fix them at room temperature for 5 min in a fixation solution containing 2% formaldehyde and 0.2% glutaraldehyde.

(B) Stain with freshly prepared SA-β-gal staining solution at 37 °C overnight.

(C) Take microscopy images and quantify the ratio of senescent myotubes by calculating the proportion of SA-β-gal positive myotubes relative to the total number of myotubes.

Step 3.5.2: Myotube diameter analysis

(A) Wash the cultured myotube cells with PBS for two times, and fix with 4% paraformaldehyde for 15 min at room temperature.

(B) Permeabilize with 0.2% Triton X-100 for 10 min and block with 10% donkey serum for 1 h at room temperature.

(C) Incubate with MyHC antibody (1:100 diluted in 1% donkey serum) at 4 °C overnight.

(D) Wash with PBS and incubate with fluorescence-labeled secondary antibody and Hoechst 33342 at room temperature for 1 h.

(E) Capture images at random with the confocal microscope, and calculate the diameters of more than 100 myotubes for each replicate. The average diameter of senescent myotubes should be decreased.

Step 3.5.3: RT-qPCR analysis

(A) Extract total RNA with TRIzol reagent.

(B) Convert 2 μg RNA to cDNAs using a GoScript Reverse Transcription System (Promega, A5001).

(C) Apply cDNA products to PCR. Primers (for humans) used for RT-qPCR are listed in Table 7. RNA expression levels of *CDKN2A*, *IL6*, *CXCL8*, *IL1A*, *IL1B*,* IFNA1*, and *IFNG* are expected to increase.

**Table 7:** Primers (for humans) used for RT-qPCR analysis

Genes	Sequence
*GAPDH*-Forward (5' - 3')	TCGGAGTCAACGGATTTGGT
*GAPDH*-Reverse (5' - 3')	TTGCCATGGGTGGAATCATA
*CDKN2A*-Forward (5' - 3')	ATGGAGCCTTCGGCTGACT
*CDKN2A*-Reverse (5' - 3')	GTAACTATTCGGTGCGTTGGG
*IL6*-Forward (5' - 3')	ACTCACCTCTTCAGAACGAATTG
*IL6*-Reverse (5' - 3')	CCATCTTTGGAAGGTTCAGGTTG
*CXCL8*-Forward (5' - 3')	ACTGAGAGTGATTGAGAGTGGAC
*CXCL8*-Reverse (5' - 3')	AACCCTCTGCACCCAGTTTTC
*IL1A*-Forward (5' - 3')	TGTAAGCTATGGCCCACTCCA
*IL1A*-Reverse (5' - 3')	AGAGACACAGATTGATCCATGCA
*IL1B*-Forward (5' - 3')	CTCTCTCCTTTCAGGGCCAA
*IL1B*-Reverse (5' - 3')	GAGAGGCCTGGCTCAACAAA
*IFNA1*-Forward (5' - 3')	GCCTCGCCCTTTGCTTTACT
*IFNA1*-Reverse (5' - 3')	CTGTGGGTCTCAGGGAGATCA
*IFNG*-Forward (5' - 3')	TCGGTAACTGACTTGAATGTCCA
*IFNG*-Reverse (5' - 3')	TCGCTTCCCTGTTTTAGCTGC

Step 3.5.4: Western blotting analysis

(A) Lyse myotube cells by heating in lysis buffer at 105 °C for 10 min.

(B) Perform protein quantification using the BCA quantification kit.

(C) Subject protein samples to SDS–PAGE and conduct electrotransfer to PVDF membranes.

(D) Block membranes with 5% milk in TBST (20 mmol/L Tris-HCl, pH 7.5, 140 mmol/L NaCl, 0.1% Tween-20) for 1 h, and incubate with primary antibodies at 4 °C overnight.

(E) Block with HRP-conjugated secondary antibodies at room temperature for 1 h after washing with TBST.

(F) Using a ChemiDoc XRS + System with Image Lab software to image the Western blots.

(G) Perform quantification of the indicated protein bands with ImageJ. Protein levels of P16, P21, and muscular dystrophy-related proteins MuRF1and FBX32 are expected to increase in senescent myotubes.

Step 3.5.5: ELISA analysis

(A) Collect cell medium and filter it with a 0.2-μm filter.

(B) Incubate in the 96-well plate with either anti-IL6 or CXCL8 antibodies according to the manufacturer’s instructions.

(C) Add the standards and cell medium to the wells pre-coated with antibody and incubate for 2 h.

(D) Wash four times and then incubate with Avidin-HRP for 1 h.

(E) Wash four times and add TMB substrate solution to stop the reaction.

(F) Measure the plate at 450 nm and normalize IL6 and CXCL8 levels to the corresponding cell numbers. The expression levels of IL6 and CXCL8 in the culture medium of senescent myotubes are expected to be increased.

**[TROUBLESHOOTING]**

Troubleshooting suggestion can be find in Table 8.

**Table 8:** Troubleshooting table

Description	Possible reason	Suggestion
No sufficient hMPCs to initiate subsequent differentiation	Pluripotent cells might contain a substantial population of differentiated cells.	1. Pluripotent cells must be homogeneous without a substantial population of differentiated cells. 2. The starting population of hESCs or iPSCs for differentiation toward hMPCs should be increased.
Low differentiation efficiency of myotube	The hMPC differentiation potential is limited.	At the beginning of myotube differentiation, early-passage hMPCs with higher differentiation potential should be used.
Failure to observe senescence-related phenotypes	Myotube cells are treated and/or collected at inappropriate time points.	It’s necessary to evaluate the senescence phenotypes at multiple time points after initiation of myotube differentiation.

## ADVANTAGES AND LIMITATIONS OF THE PROTOCOL

This tutorial protocol provides a detailed guide to obtaining myotubes for phenotypical and mechanistic studies of human myotube senescence and for drug screening to identify agents that could alleviate skeletal muscle aging. However, several limitations remain. First, to induce myotube differentiation, we deliver the master regulatory factor MyoD into hMPCs through an integrating lentiviral vector that may introduce genomic instability. Secondly, although induced myotube cells recapitulate a series of senescence-associated phenotypes *in vitro*, it remains a model system that may not fully mimic skeletal muscle aging *in vivo.* Thirdly, two-dimensional cell culture systems lack the structure of skeletal (myotube bundles) and interactions with other cells. The development of 3D models or engineered muscle cell models using biological materials and specialized equipment may facilitate studies of cell-cell interactions, cell-matrix interactions, cell mechanical properties, and so on (Faustino Martins *et al.* 2020; Juhas *et al.* 2018; Pinton *et al.* 2023). Collectively, our protocol provides a valuable platform for investigating mechanisms underlying human skeletal muscle aging and as such stands to facilitate the development of therapeutic strategies against skeletal muscle aging and related disorders.

## Conflict of interest

Qian Zhao, Ying Jing, Shuai Ma, Weiqi Zhang, Jing Qu, Si Wang and Guang-Hui Liu declare that they have no conflict of interest.
